# Association between illness severity and timing of initial enteral feeding in critically ill patients: a retrospective observational study

**DOI:** 10.1186/1475-2891-11-30

**Published:** 2012-05-03

**Authors:** Hsiu-Hua Huang, Chien-Wei Hsu, Shiu-Ping Kang, Ming-Yi Liu, Sue-Joan Chang

**Affiliations:** 1 Department of Life Sciences, College of Bioscience and Biotechnology, National Cheng Kung University, No.1, University Rd., Tainan City 701, Taiwan; 2 Department of Food and Nutrition, Taipei Veterans General Hospital, No.201, Sec. 2, Shipai Rd., Beitou Dist., Taipei City 11217, Taiwan; 3 Medicine Department, School of Medicine, National Yang-Ming University, No.155, Sec.2, Linong Street, Beitou Dist., Taipei City 11221, Taiwan; 4 Intensive Care Unit, Department of Medicine, Kaohsiung Veterans General Hospital, No. 386, Ta-Chung First Rd., Zouying Dist., Kaohsiung City 81362, Taiwan; 5 Department of Nursing, Kaohsiung Veterans General Hospital, No. 386, Ta-Chung First Rd., Zouying Dist., Kaohsiung City 81362, Taiwan; 6 Department of Nutrition, SinLau Hospital, No. 57, Sec. 1, Dongmen Rd., Tainan City 70142, Taiwan

**Keywords:** Severity of illness, Early enteral feeding, Late enteral feeding, Critical illness

## Abstract

**Background:**

Early enteral nutrition is recommended in cases of critical illness. It is unclear whether this recommendation is of most benefit to extremely ill patients. We aim to determine the association between illness severity and commencement of enteral feeding.

**Methods:**

One hundred and eight critically ill patients were grouped as “less severe” and “more severe” for this cross-sectional, retrospective observational study. The cut off value was based on Acute Physiology and Chronic Health Evaluation II score 20. Patients who received enteral feeding within 48 h of medical intensive care unit (ICU) admission were considered early feeding cases otherwise they were assessed as late feeding cases. Feeding complications (gastric retention/vomiting/diarrhea/gastrointestinal bleeding), length of ICU stay, length of hospital stay, ventilator-associated pneumonia, hospital mortality, nutritional intake, serum albumin, serum prealbumin, nitrogen balance (NB), and 24-h urinary urea nitrogen data were collected over 21 days.

**Results:**

There were no differences in measured outcomes between early and late feedings for less severely ill patients. Among more severely ill patients, however, the early feeding group showed improved serum albumin (p = 0.036) and prealbumin (p = 0.014) but worsened NB (p = 0.01), more feeding complications (p = 0.005), and prolonged ICU stays (p = 0.005) compared to their late feeding counterparts.

**Conclusions:**

There is a significant association between severity of illness and timing of enteral feeding initiation. In more severe illness, early feeding was associated with improved nutritional outcomes, while late feeding was associated with reduced feeding complications and length of ICU stay. However, the feeding complications of more severely ill early feeders can be handled without significantly affecting nutritional intake and there is no eventual difference in length of hospital stay or mortality between groups. Consequently, early feeding shows to be a more beneficial nutritional intervention option than late feeding in patients with more severe illness.

## Introduction

Critical illness changes substrate metabolism, thereby altering body compositions and worsening clinical outcomes [1]. Intensive care unit (ICU) patients are susceptible to malnutrition, immune dysfunction, severe infections, multiple organ dysfunction, and death [2,3].

Early enteral feeding improves clinical outcomes, reduces gastric intolerance, and promotes early reestablishment of gastroduodenal motility [4,5]. Patients experiencing early enteral feeding (within 24 to 48 h following ICU admission) demonstrate reduced gut permeability and cytokine release, compared to late enteral feeding patients (after 72 h) [6]. However, Ibrahim et al. observed that the administration of early enteral nutrition to mechanically ventilated medical patients is associated with more severe infectious complications and prolonged ICU stays [7]. Minard et al. stated that patients with severe closed-head injuries demonstrated no differences in length of stay or infectious complications in early vs. delayed feeding [8]. Therefore, the consistency of the current medical evidence from systematic reviews may be insufficient to convince clinicians to aggressively provide early feeding in more severely ill patients [9]. Although many studies have investigated the timing of enteral nutrition in critical illness, its effects on clinical outcomes in patients with varied illness severity have not been fully examined.

This study aims to determine the association between illness severity and commencement of enteral feeding. The primary outcome measures are clinical outcomes while secondary measures are nutritional outcomes. The study investigates the association between illness severity and feeding complications, length of ICU stay, length of hospital stay, ventilator-associated pneumonia (VAP), hospital mortality rate, serum albumin, serum prealbumin, nitrogen balance (NB), and nutritional intake over a 21-d study period in critically ill patients receiving enteral feeding within or after 48 h of ICU admission.

## Materials and Methods

### Subjects and Study Design

This retrospective observational study was conducted between January 2005 and December 2006 at Kaohsiung Veterans General Hospital. Study protocol was conducted in accordance with the ethical standards of the World Medical Association Declaration of Helsinki and approved by the hospital’s Research Ethics Committee. All patients consecutively admitted to the medical ICU were enrolled unless enteral feeding was contraindicated. Contraindications included: paralytic ileus, intestinal obstruction, intractable vomiting, persistent watery diarrhea, active gastrointestinal (GI) bleeding, short bowel syndrome or severe acute pancreatitis. Patients intravenously supplemented with fat emulsion, amino acids or albumin during the study period were also excluded. After admission, patients were administered nasogastric or nasoduodenal feeding tubes (12Fr enteral feeding tube, Flexiflo, Abbott, Chicago, IL) with full-strength isotonic formula (Jevity, Abbott Laboratories, Ontario, Canada), starting at 20 mL/h, and increasing by 20 mL/h every 4 h to satisfy energy and protein requirements recommended by a clinical dietitian based on the Ireton-Jones equation: EEE (v) = 1784 − 11(A) + 5(W) + 244(S) + 239(T) + 804(B) − 609(O); REI = EEE × (1.0-1.5), where EEE = estimated energy expenditure (kcal/day), v = ventilator dependent, A = age (yr), W = body weight (kg), S = sex (male = 1, female = 0), diagnosis of T = trauma, B = burn, O = obesity (if present = 1, absent = 0), REI = recommended energy intake, and Canadian clinical practice guidelines for critically ill adult patients [10]. Daily recommended energy and protein requirements ranged from 25–30 kcal/kg and 1.2–1.5 g/kg ideal body weight. All patients were fed with heads elevated 30-45 ° during feeding and for 1 h after feeding. Residual was checked every 4 h and feeding was withheld for 1 h if residual volume was over 250 ml. The nurses interrupted enteral feeding in cases of: overt regurgitation or aspiration; residual volume over 500 mL; residual volume between 250 and 500 mL with abdominal distention, nausea or vomiting [11]. Residual was rechecked before reinitiating feeding. Once the residual volume was lower than 250 mL and patients showed no abdominal distention, nausea or vomiting, tube feeding was restarted at a rate of 20 mL/h and increased by 20 mL/h every 4 h until the caloric target was achieved. Patients were monitored for up to 21 days or observations were closed if they expired or were transferred to an ordinary ward.

### Definitions and outcome measures

The timing of initial enteral feeding was determined by the ICU team following Society of Critical Care Medicine and American Society for Parenteral and Enteral Nutrition guidelines. Feeding commencement at less than 48 h after ICU admission was considered early feeding while over 48 h was considered late feeding. Illness severity was determined by Acute Physiology and Chronic Health Evaluation (APACHE) II scores [12,13]. Previous studies have indicated that the optimal cutoff estimate for APACHE II scoring in predicting ICU mortality is 20; patients with APACHE II scores ≥ 20 on ICU admission demonstrate a significantly higher fatality risk than those with scores < 20 [14-16]. Based on this information, this study groups eligible patients as ‘less severe’ (APACHE II score < 20) or ‘more severe’ (APACHE II score ≥ 20) and then sub-groups these categories into early or late feeding groups according to when enteral feeding started. In clinical practice, NB was calculated by: daily protein intake (g) ÷ 6.25 - (24-h UUN (g) + 4 g obligatory loss) and can be used to estimate the magnitude of stress response as reflected by the catabolic rate [17]. There were no massive extra renal nitrogen losses (inflammatory bowel disease, GI fistulae, extensive bed sores, burn exudates) in our patients. Because there is no objective indicator of GI function in critically ill patients [18,19], we assessed GI dysfunction based on the clinical assessments of four tube feeding complications: gastric retention, vomiting, diarrhea, and GI bleeding. Gastric retention was defined as a residual volume >250 mL [6,18]; vomiting as feeding formula found in the pharynx or mouth; diarrhea as ≥3 bowel movements or >200 mL watery stool/day in patients who had not consumed laxatives or hyperosmolar medications in the preceding 24 h; and GI bleeding as presence of hematemesis, melena, bloody stool, or coffee grounds-like material in the feeding tubes. A feeding complication episode was based on the first appearance of symptoms until the symptoms subsided. Length of ICU stay equalled ICU admission until transfer out of ICU. VAP was diagnosed by two pulmonologists using a modified National Nosocomial Infections Surveillance system [20]. Hospital mortality was defined as death while hospitalized. Caloric and protein intake were calculated from the amount of administered formula as specified in the medical records and nurse files. The percentage of target caloric and protein intake achieved was calculated as: mean % of target caloric (protein) intake = Σ [each day’s caloric (protein) intake ÷ recommended daily caloric (protein) requirement] ÷ number of study days.

### Data collection

Basic patient characteristics (age, sex, height, weight), ICU admission and transfer-out dates, diagnosis and APACHE II score on ICU admission, survival or death at hospital discharge, VAP, medications administered, start and end dates of tube feeding, daily caloric and protein intake, and clinical assessments of tube feeding complications (gastric retention/vomiting/diarrhea/GI bleeding) were recorded. Blood and 24 h urine samples were collected on feeding days 1, 4, 7, 14, and 21 for laboratory measurements of serum albumin, serum prealbumin, and 24-h urinary urea nitrogen (UUN).

### Statistical analysis

All statistical analyses were performed using SPSS version 15.0 (SPSS Inc., Chicago, IL, USA) and Excel 2003 (Microsoft, Redmond, WA, USA). Distributions of baseline characteristics, differences in clinical outcomes, biochemical values and nutritional intake between groups were compared using Student’s *t*-test for normally distributed continuous variables or Mann–Whitney *U*-test for non-normally distributed continuous variables. Multiple linear regression and logistic regression were used to assess initial enteral feeding time’s effects on measured outcomes after adjusting for potential confounders. Two-tailed p-values < 0.05 were considered significant. Values are presented as means ± standard deviation.

## Results

### Patients

A total of 108 newly admitted ICU patients qualified for the trial. Of these, 40 patients (43.5 %) received early enteral feeding with 14 assigned to ‘less severe’ and 26 assigned to ‘more severe’. The other 68 patients (56.5 %) received late enteral feeding with 33 assigned to ‘less severe’ and 35 assigned to ‘more severe’. Demographic and clinical characteristics of the patients are shown in Table 1. The early feeding patients were significantly older than those in the late feeding group, but these differences did not exist between groups broken-down by illness severity. Notably, early feeding patients had a significantly higher incidence of antibiotic use.

**Table 1:** Demographic and clinical characteristics of the patients categorized by timing of feeding initiation and break down by illness severity

**Characteristic**			**APACHE II <20**		**APACHE II ≥20**	
	**Early**	**Late**				
			**(n = 47)**		**(n = 61)**	
	**(n = 40)**	**(n = 68)**				
			**Early**	**Late**	**Early**	**Late**
			**(n = 14)**	**(n = 33)**	**(n = 26)**	**(n = 35)**
**Gender (M/F), n**	M 27	M 53	M 8	M 26	M 19	M 27
	F 13	F 15	F 6	F 7	F 7	F 8
**Age (y)**	72.8 ± 11.1^†^	67.5 ± 15.9*	64.1 ± 13.6	62.3 ± 15.2	77.5 ± 5.5	72.4 ± 15.2
**Body mass index (kg/m**^**2**^**)**	23.1 ± 4.4	23.5 ± 5.5	25.4 ± 4.6	24.5 ± 6.1	21.8 ± 3.8	22.6 ± 4.8
**Diagnosis at ICU admission, n**						
Cardiovascular	7	12	2	5	5	7
Respiratory	20	30	7	15	13	15
Gastrointestinal	3	9	1	3	2	6
Neurological	3	10	1	6	2	4
Sepsis	4	5	1	3	3	2
Others	3	2	2	1	1	1
**Mechanical ventilation, n (%)**	40 (100)^†^	68 (100)	14 (100)	33 (100)	26 (100)	35 (100)
**Admission to feeding initiation (h)**	45.6 ± 7.3	109.4 ± 47.7 ***	48.0 ± 0.0	109.1 ± 46.1 ***	44.3 ± 8.8	109.7 ± 53.4 ***
**Enteral feeding route, n**						
NG tube feeding	23	35	6	18	17	17
ND tube feeding	17	33	8	15	9	18
**Recommended energy requirement (kcal/d)**	1718 ± 174	1757 ± 184	1721 ± 193	1755 ± 172	1715 ± 167	1760 ± 197
**Recommended protein requirement (g/d)**	69.5 ± 12.4	69.1 ± 12.4	71.4 ± 12.3	70.6 ± 11.6	68.4 ± 12.6	67.7 ± 13.0
**APACHE II score at ICU admission**	21.4 ± 6.2	20.4 ± 7.1	15.3 ± 3.2	14.6 ± 2.8	24.7 ± 4.8	25.9 ± 5.4
**Patients taking prokinetic agents, n (%)**	13 (32.5)	23 (33.8)	3 (21.4)	12 (36.4)	10 (38.5)	11 (31.4)
**Patients taking PPIs or H**_**2**_**antagonists, n (%)**	15 (37.5)	17 (25.0)	4 (28.6)	7 (21.2)	11 (42.3)	10 (28.6)
**Patients taking antibiotics, n (%)**	39 (97.5)	54 (79.4) **	14 (100)	26 (78.8)**	25 (96.2)	28 (80)*

APACHE, Acute Physiology and Chronic Health Evaluation; ICU, intensive care unit; NG, nasogastric; ND, nasoduodenal; PPI, proton pump inhibitor.

^†^Data are expressed as mean ± SD, number of patients, or percentage in parentheses. Student’s *t*-test for differences between early and late feeding groups, Mann–Whitney *U*-test for differences between early and late feeding groups categorized by APACHE II <20 and APACHE II ≥20. Single asterisk (*) indicates p < 0.05, double asterisks (**) indicate p < 0.01, and triple asterisks (***) indicate p < 0.001.

### Clinical outcomes

#### Feeding complications

The frequency of feeding complications was not statistically different between early and late feeding groups. However, more severely ill early feeding group patients experienced significantly higher feeding complications than their counterparts of the late feeding group (Table 2). Diarrhea and GI bleeding were significantly higher in early feeders among the more severely ill (Table 3). After adjusting for gender, age and illness severity, early enteral feeding had positive effects on feeding complications (Table 4).

**Table 2:** Differences in measured outcomes between early and late feeding groups and break down by illness severity

**Measured outcomes**	**Early**	**Late**	**APACHE II <20**		**APACHE II ≥20**	
	**(n = 40)**	**(n = 68)**	**(n = 47)**		**(n = 61)**	
			**Early**	**Late**	**Early**	**Late**
			**(n = 14)**	**(n = 33)**	**(n = 26)**	**(n = 35)**
**Clinical outcomes**						
Frequency of feeding complications (times/d)	0.11 ± 0.18^†^	0.08 ± 0.18	0.05 ± 0.12	0.10 ± 0.24	0.14 ± 0.20	0.05 ± 0.11**
VAP (%, n)	32.5 (13/40)	13.8 (9/65)*	28.6 (4/14)	9.1 (3/33)	34.6 (9/26)	18.8 (6/32)
Length of ICU stay (d)	21.1 ± 14.3	15.0 ± 7.7*	16.4 ± 13.3	15.9 ± 9.1	23.6 ± 14.4	14.2 ± 6.0**
Length of hospital stay (d)	37.0 ± 26.2	33.07 ± 23.1	24.1 ± 17.4	34.8 ± 27.3	43.9 ± 27.8	31.4 ± 18.5
Hospital mortality (%, n)	45.0 (18/40)	36.8 (25/68)	35.7 (5/14)	18.2 (6/33)	50.0 (13/26)	54.3 (19/35)
**Nutritional outcomes**						
% of target caloric intake	86.9 ± 21.7	82.2 ± 26.3	85.3 ± 19.5	84.4 ± 24.4	87.8 ± 23.1	80.1 ± 28.2
% of target protein intake	89.6 ± 26.6	85.7 ± 32.5	84.9 ± 18.7	84.6 ± 28.6	92.1 ± 30.1	86.7 ± 36.1
Albumin on 7th feeding day (g/L)	20.9 ± 4.8	20.0 ± 5.1	21.4 ± 5.8	21.9 ± 3.8	20.7 ± 4.5	18.2 ± 5.6*
	(n = 35)	(n = 47)	(n = 10)	(n = 23)	(n = 25)	(n = 24)
Prealbumin on 4th feeding day (mg/dL)	16.3 ± 13.3	14.1 ± 8.3*	16.1 ± 8.6	16.2 ± 7.3	13.3 ± 3.5	12.1 ± 8.8*
	(n = 40)	(n = 65)	(n = 14)	(n = 32)	(n = 26)	(n = 33)
Prealbumin on 7th feeding day (mg/dL)	15.4 ± 5.4	14.0 ± 7.7	14.9 ± 7.0	16.3 ± 8.5	15.6 ± 4.7	11.9 ± 6.5*
	(n = 34)	(n = 46)	(n = 10)	(n = 22)	(n = 24)	(n = 24)
NB on 7th feeding day (g/d)	−8.0 ± 7.9	−3.7 ± 5.3**	−10.2 ± 7.5	−3.6 ± 5.3**	−7.1 ± 8.0	−3.7 ± 5.5
	(n = 34)	(n = 45)	(n = 10)	(n = 23)	(n = 24)	(n = 22)
NB on 14th feeding day (g/d)	−6.8 ± 5.9	−1.6 ± 4.6**	−5.1 ± 5.7	−2.4 ± 5.4	−7.5 ± 6.0	−0.8 ± 3.8**
	(n = 19)	(n = 19)	(n = 5)	(n = 9)	(n = 14)	(n = 10)
24-h UUN on 14th feeding day (g/d)	12.8 ± 4.3	8.3 ± 4.9**	12.7 ± 7.0	9.3 ± 5.0	12.8 ± 3.2	7.4 ± 4.8**
	(n = 19)	(n = 19)	(n = 5)	(n = 9)	(n = 14)	(n = 10)
24-h UUN on 21st feeding day (g/d)	9.5 ± 2.9	5.5 ± 3.0*	12.4 ± 3.9	7.6 ± 1.4	8.6 ± 2.1	2.4 ± 0.2*
	(n = 13)	(n = 5)	(n = 3)	(n = 3)	(n = 10)	(n = 2)

APACHE, Acute Physiology and Chronic Health Evaluation; VAP, ventilator-associated pneumonia; ICU, intensive care unit. ^†^Data are expressed as mean ± SD, percentage, and number of patients in parentheses. Student’s *t*-test for differences between early and late feeding groups, and Mann–Whitney *U*-test for differences between both feeding groups categorized by APACHE II <20 and APACHE II ≥20. Single asterisk (*) indicates p < 0.05, double asterisks (**) indicate p < 0.01.

**Table 3:** Differences in feeding complications between early and late feeding groups categorized by illness severity

**Feeding complications**	**APACHE II <20**		**APACHE II ≥20**	
	**(n = 47)**		**(n = 61)**	
	**Early**	**Late**	**Early**	**Late**
	**(n = 14)**	**(n = 33)**	**(n = 26)**	**(n = 35)**
Diarrhea (%, n)	14.3 (2/14)^†^	24.2 (8/33)	42.3 (11/26)	11.4 (4/35)**
GI bleeding (%, n)	7.7 (1/13)	18.2 (6/33)	30.8 (8/26)	5.7 (2/35)**
Vomiting (%, n)	7.1 (1/14)	3.0 (1/33)	11.5 (3/26)	8.8 (3/34)
Gastric retention (%, n)	0 (0/14)	0 (0/33)	3.8 (1/26)	5.7 (2/35)

APACHE, Acute Physiology and Chronic Health Evaluation; GI, gastrointestinal. ^†^Data are expressed as percentage and number of patients in parentheses. Mann–Whitney *U*-test for differences between early and late feeding groups categorized by APACHE II <20 and APACHE II ≥20. Double asterisks (**) indicate p < 0.01.

**Table 4:** Effects of the timing of enteral feeding initiation (early feeding) on different clinical and nutritional outcomes

**Measured outcomes**	**APACHE II <20**			**APACHE II ≥20**		
	**(n = 47)**			**(n = 61)**		
**Multiple linear regression**^†^	**β**	**S.E.**	**p**	**β**	**S.E.**	**p**
**Clinical outcomes**						
Frequency of feeding complications (times/d)	−0.045	0.070	0.526	0.084	0.042	0.05
Length of ICU stay (d)	−0.003	3.350	0.999	9.401	2.785	0.001
Length of hospital stay (d)	−10.731	8.187	0.197	10.654	6.155	0.089
**Nutritional outcomes**						
% of target caloric intake	−0.230	7.832	0.977	4.509	6.709	0.504
% of target protein intake	−0.444	8.898	0.960	1.412	8.777	0.873
Albumin on 7^th^ feeding day (g/L)	92.569	183.796	0.618	220.398	153.507	0.158
Prealbumin on 4^th^ feeding day (mg/dL)	−0.574	2.527	0.821	1.961	1.874	0.300
Prealbumin on 7^th^ feeding day (mg/dL)	−1.882	2.916	0.524	3.110	1.688	0.072
24-h UUN on 14^th^ feeding day (g/d)	6.455	4.435	0.180	5.343	1.730	0.006
24-h UUN on 21^st^ feeding day (g/d)	5.398	0.516	0.061	6.816	1.864	0.008
NB on 7^th^ feeding day (g/d)	−8.615	2.219	0.001	−3.687	2.116	0.089
NB on 14^th^ feeding day (g/d)	−5.954	4.407	0.210	−7.126	2.194	0.004
**Logistic regression**^†^	**Adjusted OR**	**95 % CI**	**p**	**Adjusted OR**	**95 % CI**	**p**
**Clinical outcomes**						
VAP	2.697	0.38-19.0	0.319	2.062	0.56-7.56	0.275
Hospital mortality	0.400	0.09-1.77	0.227	1.083	0.36-3.29	0.888
Diarrhea	0.519	0.09-2.99	0.463	9.455	1.81-49.44	0.008
GI bleeding	0.433	0.03-6.58	0.547	7.075	1.24-40.32	0.028

APACHE, Acute Physiology and Chronic Health Evaluation; NB, nitrogen balance; UUN, urinary urea nitrogen; VAP, ventilator-associated pneumonia; ICU, intensive care unit.

^†^Multiple linear or logistic regression for examining the effects of the timing of enteral feeding initiation (X) on measured outcomes (Y) after adjusting for gender, age and illness severity. Coding for the timing of enteral feeding initiation (X): early feeding = 1, late feeding = 0.

#### VAP

The incidence of VAP was significant higher in the early feeding group; however, there was no significant difference between both groups by illness category (Table 2) nor was VAP related to enteral feeding commencement (Table 4).

#### Lengths of ICU stay and hospital stay

The early feeding group experienced longer ICU stays. However, these differences only existed among more but not less severely ill patients (Table 2). After adjusting for gender, age and severity of illness, early enteral feeding was associated with increased length of ICU stay among the more severely ill patients (Table 4). There were no differences in length of hospital stay (Table 2).

#### Hospital mortality

There were no differences between both groups in either illness category (Table 2). Timing of enteral feeding initiation was not associated with hospital mortality (Table 4). However, after adjusting for gender, age and timing of enteral feeding initiation, illness severity had an effect on mortality rate. Patients with higher APACHE II scores risked higher hospital mortality (adjusted OR = 0.897, 95 % CI = 0.835-0.964, p = 0.003).

### Nutritional outcomes

#### Nutritional intake

There were no differences in caloric and protein intake between both feeding groups for less or more severely ill patients (Tables 2 and 4). We observed a mean of 80 % above the caloric intake goal for both feeding groups (Table 2).

#### Serum albumin and prealbumin

The more severely ill early feeding patients showed significantly higher serum albumin levels than those of the late feeding group on feeding day 7; less severely ill patients showed no differences (Table 2). The commencement time of enteral feeding had no effect on serum albumin levels after adjusting for gender, age and illness severity (Table 4). Similarly, serum prealbumin levels were significantly higher in the early feeding group on feeding days 4 and 7 (Table 2) in the more severely ill patients; less severely ill patients showed no differences between groups. After adjusting for gender, age and illness severity, early feeding was not associated with increased serum prealbumin levels in more severely ill patients (Table 4).

#### NB and 24-h UUN

The NB and 24-h UUN in both groups significantly differed on feeding days 7, 14 or 21 among more but not less severely ill patients (Table 2). Adjusting for gender, age and illness severity, the timing of enteral feeding initiation had effects on NB and 24-h UUN (Table 4). Patients with higher APACHE II scores in the early feeding group experienced more nitrogen loss and worsened NB.

### Post-hoc power

Post-hoc power analyses were performed using G*Power 3.1 software [21] to determine whether the sample size could give acceptable results. Among less severely ill patients, power was insufficient to analyze the differences between early and late feeding groups in terms of clinical outcomes and nutritional outcomes. However, for these patients, multiple linear regression and logistic regression analyses verified the timing of enteral feeding initiation had no effect on measured outcomes after adjusting for confounding factors (Table 4). On the other hand, in the case of APACHE II ≥20 patients, our study had sufficient power to analyze length of ICU stay (power = 0.9), NB on feeding day 14 (power = 0.85) and 24-h UUN on feeding days 14, 21 (power = 0.8, 0.99); but insufficient power for analyzing the feeding complications (power = 0.6), serum albumin on feeding day 7 (power = 0.6) and serum prealbumin on feeding days 4, 7 (power = 0.13, 0.72).

## Discussion

The early feeders among the more severely ill patients experienced significantly more frequent feeding complications than late feeders, but their caloric and protein intake did not differ from the other groups. This was due to their major feeding complications being diarrhea and/or GI bleeding and not gastric retention or vomiting, which would significantly affect nutritional intake. Severely ill patients commonly develop GI problems such as mucosal damage, motility disturbances, and hypoalbuminemia-related mucosal edema due to severe physiological stress [22,23]. Therefore, we inferred that the higher incidence of diarrhea might have been due to early aggressive feeding placing stress on damaged mucosa and/or a higher usage of antibiotics in these patients. However, diarrhea among the more severely ill early feeders was not severe and it subsided after adjusting the feeding rate and/or administration of anti-diarrhea medicines. Additionally, the higher incidence of GI bleeding among these patients is related to stress ulcers and not active bleeding. GI bleeding subsided after controlling shock or use of medications such as proton pump inhibitors or histamine type 2 receptor antagonists. Consequently, as we were able to control diarrhea and GI bleeding, neither significantly affected nutritional intake. These results, however, are inconsistent with previous reports of early feeding improving GI function [4,6]. Critically ill patients suffer from a combination of physiological disturbances likely to influence GI function [22,24]. Our results show an association between illness severity and enteral feeding commencement. In more severely ill patients, feeding within 24 to 48 h of ICU admission may be too early as it can cause further stress to the GI tract, and result in diarrhea and stress-induced ulcers.

We also observed that the higher the APACHE II score, the longer the ICU stay for early feeders after adjusting for confounding factors. These results are consistent with Ibrahim et al., who observed greater incidence of diarrhea and longer ICU stays among early feeders [7]. We hypothesize that the greater incidence of feeding complications is a confounding factor increasing the length of ICU stay in the early feeding group among more severely ill patients [25].

Our observations indicate that mortality is unaffected by enteral feeding commencement time. The meta-analysis study conducted by Marik and Zaloga also found no relationship between early enteral nutrition and decreased mortality [26]. Expectably, our study demonstrates that illness severity governs the mortality rate and neither late enteral feeding nor early enteral feeding reduces the mortality rate.

Clinically, serum albumin level most likely acts as a prognostic rather than nutritional indicator [27]. Previous studies have indicated that inflammatory mediators and cytokines released during injury are major contributors in lowering serum albumin and prealbumin levels [28,29]. Serum prealbumin is more sensitive to changes in protein-energy status than serum albumin is, and its concentration reflects recent dietary intake rather than an overall nutritional status [30]. In critical illness, hypoalbuminemia and hypoprealbuminemia are very common and inversely related to C-reactive protein [30]. Therefore, increases in these two serum protein levels (in response to enteral feeding on days 4 and 7) only in the case of more severely ill early feeders might relate to early feeding inducing the release of trophic endogenous agents and the inhibitory effects of inflammatory mediators and cytokines released during severe illness [26,31].

Negative NB indicates inadequate protein intake or excessive catabolism. We observed no differences in protein intake between both feeding groups but significantly higher 24-h UUN losses in more severely ill early feeders. This implies that levels of stress are higher among these patients. Briassoulis et al. demonstrated that severity of illness independently contributes to negative NB status during acute stress phases [32]. As disease becomes more severe, more stress hormones are secreted, leading to greater GI disturbances and nitrogen loss [33]. Therefore, the lower 24-h UUN loss and better NB in the more severely ill of late feeding group is more likely due to improved metabolic stress.

This study has important strengths. Firstly, it is observational and feeding commencement was decided solely by the ICU team in accordance with actual treatment protocol. This connotes the de facto aspects of intensive care. Second, our 21-d study period is longer than those of previous studies. There were also limitations in our study. First, the study population was within a single medical ICU, meaning generalizations must be treated cautiously. Second, the sample size of the early feeding group limits the study’s power to analyze the measured outcomes. However, the results of multiple linear regression and logistic regression analyses strongly support our findings eliminating the issue of power insufficiency. Further, larger randomized sample-sizes, controlled trials with mixed ICU patients, data analyzed on tertiles, quartiles or quintiles of APACHE II scores and enteral feeding commencement time are required to fully investigate the optimal timing of initial enteral feeding in managing patients with varied illness severity.

## Conclusions

Genetic polymorphisms, underlying pathology, and patient heterogeneity limit the extent to which nutritional intervention can be standardized. Our study demonstrates a relationship exists between illness severity and enteral feeding commencement time influencing clinical outcomes. For more severely ill patients, early feeding is associated with improved nutritional outcomes whereas late feeding is associated with reduced feeding complications and shorter ICU stays. Notably, the feeding complications of more severely ill early feeders can be handled without significantly affecting nutritional intake. Additionally, the study shows there is no eventual difference in length of hospital stay or mortality although such patients experience longer ICU stays. Consequently, early feeding shows to be a more beneficial nutritional intervention option than late feeding in patients with more severe illness.

## Abbreviations

ICU: Intensive care unit; GI: Gastrointestinal; APACHE: Acute physiology and chronic health evaluation; VAP: Ventilator-associated pneumonia; UUN: Urinary urea nitrogen; NB: Nitrogen balance; BMI: Body mass index; PPI: Proton pump inhibitor.

## Competing interests

The authors declare that they have no conflict of interest.

## Authors’ contributions

Author contributions to the manuscript are as follows: H H-H performed statistical analyses, interpreted the results and wrote the manuscript; C S-J supervised the process, provided significant advice and revised the manuscript; H C-W developed the protocol, designed the experiment and provided advice; K S-P collected data and L M-Y provided consultation. All of the authors have read and approved the final manuscript.

## Authors’ information

HH-H, RD, MS, Chief of Foodservice Management Division, Department of Food and Nutrition, Taipei Veterans General Hospital (a 3000-bed teaching hospital). Board Director of Taiwan Society for Parenteral and Enteral Nutrition. Executive Supervisor of Kaohsiung Dietetic Association. HC-W, MD, Visiting Doctor of Intensive Care Unit, Department of Medicine, Kaohsiung Veterans General Hospital (a 1361-bed teaching hospital). Assistant Professor of Medicine, School of Medicine, National Yang-Ming University. KS-P, RN, MS, Registered Nurse of Nutrition Support Team, Kaohsiung Veterans General Hospital (a 1361-bed teaching hospital). L M-Y, RD, MS, Chief of Department of Nutrition, Sin-Lau Hospital (a 526-bed regional hospital). Board Director of Tainan Dietetic Association. C S-J, PhD, Distinguished Professor of Department of Life Sciences, College of Bioscience and Biotechnology, National Cheng Kung University. Secretary General of Health Food Society of Taiwan.
